# Dissecting the Genetic Basis of Flowering Time and Height Related-Traits Using Two Doubled Haploid Populations in Maize

**DOI:** 10.3390/plants10081585

**Published:** 2021-07-31

**Authors:** Lei Du, Hao Zhang, Wangsen Xin, Kejun Ma, Dengxiang Du, Changping Yu, Yongzhong Liu

**Affiliations:** 1 National Key Laboratory of Crop Genetic Improvement, Huazhong Agricultural University, Wuhan 430070, China; maizedu@163.com (L.D.); maizezh@163.com (H.Z.); sen0607@126.com (W.X.); mazhenghang@163.com (K.M.); ddx@mail.hzau.edu.cn (D.D.); 2 Maize Breeding Research Institute, Shiyan Academy of Agricultural Sciences, Shiyan 442000, China; ycpsyym2008@163.com

**Keywords:** flowering time, height trait, genotyping-by-sequencing, doubled haploid, quantitative trait locus

## Abstract

In the field, maize flowering time and height traits are closely linked with yield, planting density, lodging resistance, and grain fill. To explore the genetic basis of flowering time and height traits in maize, we investigated six related traits, namely, days to anthesis (AD), days to silking (SD), the anthesis–silking interval (ASI), plant height (PH), ear height (EH), and the EH/PH ratio (ER) in two locations for two years based on two doubled haploid (DH) populations. Based on the two high-density genetic linkage maps, 12 and 22 quantitative trait loci (QTL) were identified, respectively, for flowering time and height-related traits. Of these, ten QTLs had overlapping confidence intervals between the two populations and were integrated into three consensus QTLs (*qFT_YZ1a*, *qHT_YZ5a*, and *qHT_YZ7a*). Of these, *qFT_YZ1a*, conferring flowering time, is located at 221.1–277.0 Mb on chromosome 1 and explained 10.0–12.5% of the AD and SD variation, and *qHT_YZ5a*, conferring height traits, is located at 147.4–217.3 Mb on chromosome 5 and explained 11.6–15.3% of the PH and EH variation. These consensus QTLs, in addition to the other repeatedly detected QTLs, provide useful information for further genetic studies and variety improvements in flowering time and height-related traits.

## 1. Introduction

Maize (*Zea mays* L.) is an important food and forage crop, and an industrial raw material crop. Flowering time and height traits are important agronomical traits in maize production, and are closely linked to the switch from vegetative growth to reproductive growth [1]. However, the balance of this switch usually breaks down when the breeding process is exposed to an exotic maize germplasm source, especially tropical and sub-tropical material, which generally leads to late flowering and a greater height [2]. Therefore, flowering time and height traits are often among the main issues that must be overcome when using new maize germplasm.

Flowering time-related traits, including days to anthesis (AD), days to silking (SD), and the anthesis–silking interval (ASI), are vital to harvesting date, crop rotation schemes, and adaptation to different environments [3,4]. Previous studies have shown that these traits are highly quantitative traits in maize [5]. Map-based cloning for dissecting quantitative traits and using linked markers for improvement are an effective breeding strategy. To date, many flowering time-related major QTLs detected by QTL mapping have been proposed, such as *epc* [6], *D8idp* [7], *zfl1* [8], *conz1* [9], *ZmPR1-4* [10], *ZCN8* [11], and *eIF-4A* [12]. More recently, high-resolution populations and high-density molecular markers have been widely utilized in genetic mapping, and many novel loci for these traits have been found in maize [13,14]. Additionally, a series of genes conferring flowering time have been cloned in maize, and some molecular mechanisms have also been proposed. For example, most flowering time-related genes were confirmed to encode a CCT domain-containing protein and are relative to the photoperiod response [5,15,16,17]. Other molecular mechanisms were also found, for example, the late flowering gene *ID1* encodes a zinc finger transcription factor [18]; the early flowering gene *dlf1* encodes a leucine zipper protein [19]; and the early flowering gene *ZmSOC1* encodes a conserved MADS domain protein [20]. Other genes are regulatory elements for flowering time; for instance, the cis-acting regulatory element *Vgt1* influences the transcript expression levels of the downstream gene *ZmRap2.7* that is associated with late flowering [21], and the gene *ZmMADS1* is a positive transcriptional regulator for early flowering [22]. These quantitative trait loci (QTLs)/genes and mechanisms provide useful information for further genetic studies on flowering time.

Height traits are closely linked with planting density, lodging resistance, and biomass yield [23], and dwarf mutants are considered a decisive factor for the “green revolution” [24]. Height traits including plant height (PH), ear height (EH), and EH/PH ratio (ER) are complex traits that are controlled by multiple genes and environmental factors [25]. Due to easy measurement of height and its high heritability, a large number of these maize QTLs have been identified, most of which are clustered in at least four main regions on the genetic linkage maps: bin 1.02–1.03 [26,27,28], bin 1.04–1.06 [26,29,30,31], bin 3.05–3.07 [27,28,29,31], and bin 5.04–5.06 [30,31,32,33]. In addition, many height-related maize genes have been confirmed, and some of their molecular mechanisms have been also illustrated. Most of these genes are involved in hormone synthesis, for example, *dwarf3*, *dwarf8*, and *ZmGA3ox2* influencing gibberellin synthesis [24,34,35], and *nana plant1* impacting brassinosteroid synthesis [36]. Others are involved in hormone transport, for example, *brachytic2* influencing polar auxin transports [37]. Moreover, some are involved in signaling related to hormone synthesis, for example, *dwarf9* regulates DELLA proteins of the gibberellin signal transduction pathways [38]. There are also other types of genes that regulate plant height, such as *ZmRPH1* encoding a QWRF homolog protein in maize [39]. This useful information will be an important basis for genetic studies and variety improvements in height traits.

The half-tropical inbred line Q1 is an important germplasm source for breeding in the mountains of Southwest China. However, this line leads to late flowering and greater height in an improving variety. The objectives of this study were to exploit early flowering and dwarf alleles that are derived from temperate elite inbred lines Ye478 and Zheng58, and to identify significant QTLs for further genetic study and molecular marker-assisted (MAS) breeding. To achieve these objectives, three flowering time traits (AD, SD, and ASI) and three height traits (PH, EH, and ER) were measured or calculated in two locations for two years based on two double haploid (DH) populations. QTL analysis of these six traits was used to explore the genetic basis and to identify significant QTLs closely linked with these traits. The results will be used to understand the genetic basis of flowering time and height traits, and further provide useful alleles for MAS breeding.

## 2. Materials and Methods

### 2.1. Genetic Materials

Two maize DH populations sharing a common parent were constructed as previously described [40]. The common parent Q1 is a half-tropical inbred line that is important for hybrid corn production in the mountains of Southwest China, but shows late flowering and greater height. The other two parents, Ye478 and Zheng58, are elite inbred lines for hybrid corn production, which have been widely planted over the past two decades on the plains of China, and show early flowering and dwarfing. These three parents had no obvious diseases or pests around tasseling and silking (Figure S1). In brief, the common parent Q1 was crossed with two different parents (Ye478 and Zheng58), separately. Then, the F_1_ hybrids of Q1 × Ye478 (QY) and Q1 × Zheng58 (QZ) were subjected to haploid induction by crossing with the haploid inducer. In the following planting season, the haploid seedlings were doubled by application of 0.06% colchicine. The two DH populations were named QY and QZ and comprised 123 and 163 DH lines, respectively.

### 2.2. Phenotypic Evaluation and Statistical Analysis

The three parents and DH lines were evaluated as described previously [40] in two locations: the towns of Huangliang (31.30° N, 110.89° E, altitude: 890 m) and Zhenzi (31.45° N, 110.99° E, altitude: 1321 m), located in the city of Yichang, Hubei Province, which are typical maize-growing regions in the southwest mountains of China. The novel aspect of the current work was the new phenotype value of six flowering time and height related traits, as described below. These locations have a milder temperature and higher humidity condition during the corn growing season, and usually have no obvious diseases and pests around the tasseling period (Figure S1). In the town of Huangliang, we evaluated flowering time and height-related traits in the years of 2016 and 2017, and in the town of Zhenzi, we evaluated these traits only in 2017. In the subsequent analysis, we combined the location and year as one variable factor, namely, environment. In each environment, the parents and DH lines were planted in the field in two replicates with a completely randomized block design. Each plot consisted of one row with 10 plants per row. All field management of fertilization, irrigation, pest control, and weed management was the same as that of local fields. In practice, 75 g/m^2^ of compound fertilizer (including N, P, and K) was applied before sowing, and 20 g/m^2^ urea was replenished in the elongation period and before tasselling. We sprayed herbicide (acetochlor) once after sowing to prevent weeds and sprayed pesticides once before tasselling for pest control. Tasseling days (AD, days to 50% plants tasseling in each plot) and silking days (SD, days to 50% plants silking in each plot) were investigated, and the anthesis–silking interval (ASI) was also calculated for each plot. Five representative plants in each plot were used for collecting plant height (PH; in cm) and ear height (EH; in cm) data [31] at two weeks after tasseling, when most lines have no obvious diseases and pests. The EH/PH ratio (ER) was calculated for each plot and was transformed using the arcsine method for subsequent analysis.

Analysis of descriptive statistics (e.g., mean, range, skewness, and kurtosis) was conducted in Excel 2010. The variance of phenotypic data was estimated using variance analysis by the aov procedure in R software [41]. The model for variance analysis was Y = μ + β_G_ + γ_L_ + (γβ)_LG_ + ε_LGR_, where β_G_ represents the effect of the G^th^ DH line, γ_L_ is the effect of the L^th^ environment, (γβ)_LG_ is the corresponding interaction effect, and ε_LGR_ is the residual effect. All effects were considered to be random. The broad-sense heritability was calculated by *H^2^* = $\partial_{G}^{2}$/($\partial_{G}^{2}$ + $\partial_{GL}^{2}$/l + $\partial_{e}^{2}$/lr), where $\partial_{G}^{2}$ is genetic variance,$\;\partial_{GL}^{2}$ is genotype × environmental variance, $\partial_{e}^{2}$ is error variance, l is the number of environments, and r is the number of replicates in each environment [42]. The best linear unbiased prediction (BLUP) was calculated for each phenotype by the lme4 package [43] in R software across different environments and used for subsequent analysis.

### 2.3. Linkage Map and QTL Analysis

Two ultra-high density linkage maps of the two DH populations were developed as previously described [40]. In brief, the two DH populations were genotyped using genotyping by sequencing (GBS) technology on an Illumina 4000 platform. A total of 64,553 and 42,792 high-quality SNP and INDEL markers were obtained, respectively. Subsequently, linkage maps were constructed based on the linkage and crossover among these markers. Finally, these two linkage maps included 1101 and 1294 bins, respectively. The total map length was 1479.4 and 1872.1 cM, and the average distances between adjacent bins were 1.36 and 1.44 cM for the QY and QZ genetic linkage maps, respectively.

QTL analysis was performed using the composite interval mapping method (CIM) in Windows QTL Cartographer v2.5 [44]. The BLUP values of phenotype for each trait across the three environments were used for QTL analysis. A significant LOD threshold of a putative QTL was determined by 1000 permutation tests (*α*  =  0.05) with a step size of 1.0 cM. The LOD thresholds ranged from 2.9 to 3.2, and we used a mean value of 3 to investigate significant QTLs. The confidence interval (CI) of QTLs was defined as the 2 LOD interval flanking the QTL peak. QTLs with overlapping CIs and identical effect directions were assumed to be the same. QTL nomenclature followed the description of [45] with minor modifications: the first letter was “*q*” for QTL, followed by the trait abbreviation, a letter representing the population from which the QTL was identified (“Y” for the QY population, “Z” for the QZ population), a number indicating the chromosome, and a final letter differentiating QTLs on the same chromosome.

## 3. Results

### 3.1. Phenotypic Variation

**Figure 1:**
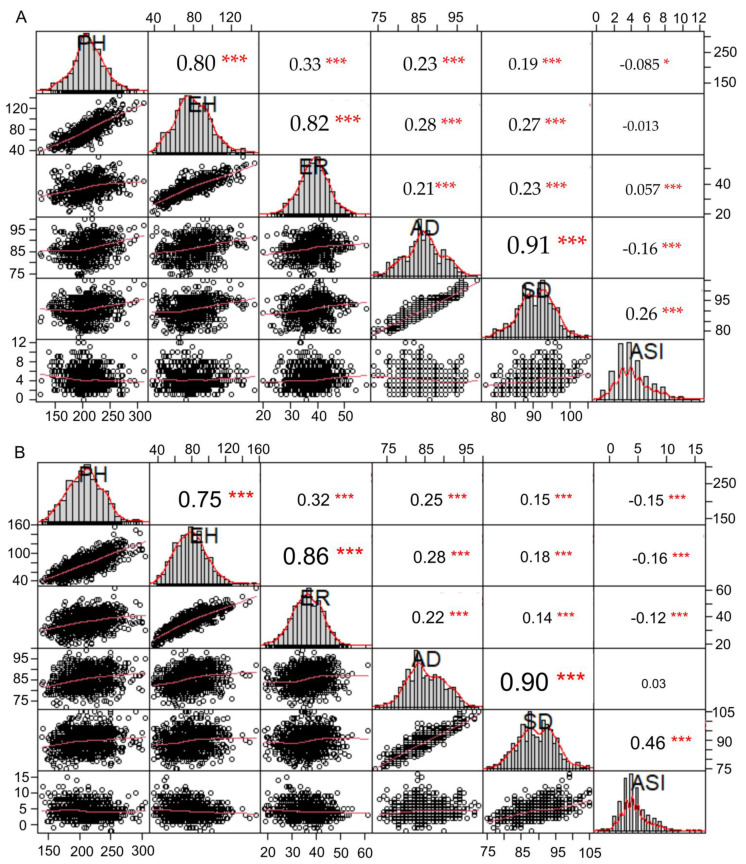
Distribution plot of phenotypic value and the Pearson correlation coefficient among different traits in the DH lines of the two DH populations: (**A**) panel represents QY population; (**B**) panel represents QZ population. On the diagonal are the bar plots of phenotypic distributions for the six traits; below the diagonal is the regression graph, and each sub-graph represents a regression of two corresponding traits on the diagonal; above the diagonal is the Pearson correlation coefficient, and each sub-graph represents a correlation coefficient of two corresponding traits on the diagonal. The “*” and “***” above the correlation coefficient represent significant *p*-value < 0.05 and 0.001, respectively.

The descriptive statistics for the six flowering time and height-related traits of the three parents and the DH lines are represented in Table 1. The parent Q1 had a later flowering time and taller height compared with the parents Ye478 and Zheng58. In addition, wide flowering time variations were observed among the DH lines in the two populations. For example, the ranges of AD and SD in the QY population were 74.0–100.0 days and 77.0–105.0 days, respectively, whereas in the QZ population, the ranges were 72.0–99.0 days and 75.0–105.0 days, respectively. Similarly, wide variations in height-related traits were also observed among the DH lines; for example, the PH ranged from 123.0 to 310.0 cm in the QY population and from 140.0 to 305.0 cm in QZ population. The EH ranged from 37.0 to 143.7 cm in the QY population and from 34.3 to 157.0 cm in the QZ population. Additionally, the frequency of phenotypic value in the two DH populations for the six traits followed an approximately normal distribution (Figure 1), indicating that these traits were controlled by QTLs. The phenotype values between PH and EH (*p* < 0.0001, 0.75–0.80), between EH and ER (*p* < 0.0001, 0.82–0.86), and between AD and SD (*p* < 0.0001, 0.90–0.91) were positively correlated in the two populations, whereas there was weaker correlativity between the flowering time and the height-related traits (Figure 1).

The genotypic variance of the AD, ASI, PH, EH, and ER traits was significant in the two populations, demonstrating real genetic differences for these six traits among DH lines in the two populations (Table S1). The difference between environments was not significant except for EH in the QY population, indicating that most of these traits were stable in each environment. The environment and genotype by environment variance were significant in the two DH populations, which could be caused by including differences of humidity, temperature, and rainfall across the environments (Table S1). Heritability for AD, AD, ASI, PH, EH, and ER ranged from 70.7 to 87.2% in the QY population, and ranged from 71.4 to 83.6% in the QZ population (Table S1). The high repeatability and heritability indicates that much of the phenotypic variance was genetically controlled in the populations and suitable for QTL mapping.

### 3.2. Identification of QTLs for Six Traits

**Figure 2:**
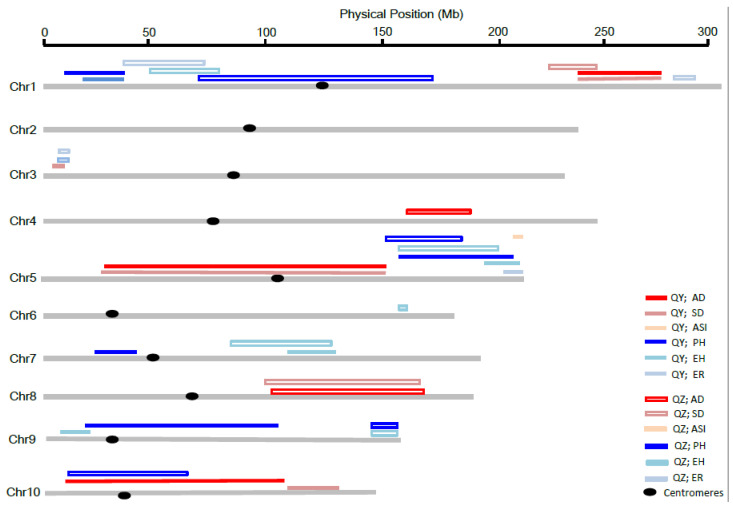
Distribution of QTLs for the six flowering time and height-related traits on the maize chromosomes. Colored lines depict QTL regions for different traits; the solid lines and hollow lines represent QTLs detected in the QY and QZ population, respectively, and the black spots represent centromeres. The red and blue colors represent flowering time and height traits, respectively, and the colors’ shading is used to distinguish the traits. The horizontal axes indicate the physical location in different chromosomes.

We conducted a QTL analysis of individual DH populations using the corresponding BLUP value across the three environments for each trait. A total of 34 QTLs associated with the six traits were identified (Table 2, Figure 2).

In the QY population, 17 QTLs were detected, with LOD scores ranging from 3.0 to 8.0. These QTLs were distributed on six chromosomes and explained 6.5 to 16.7% of the phenotypic variation. Of these QTLs, there were three, four, and one QTLs conferring the AD, SD, and ASI traits, respectively. Most of these flowering time-related QTLs decreased phenotype value when they were from Ye478, except for *qASI_Y5a*, and five of them could explain up to 10% of the phenotypic variation. In addition, four, four, and one QTLs were detected as conferring the PH, EH, and ER traits, respectively. All of these height trait-related QTLs also decreased phenotype value when they were derived from the parent Ye478, and four of them could explain up to 10% of the phenotypic variation.

In the QZ population, there also were 17 QTLs detected, with LOD scores ranging from 3.0 to 12.0. These QTLs were distributed on nine chromosomes, except for chromosome 2, and explained 4.4 to 22.0% of the phenotypic variation. Of these QTLs, there were two and two QTLs associated with the AD and SD traits, respectively, and no QTL was detected for ASI. All of these flowering time-related QTLs decreased phenotype value when they were from Zheng58, and two of them could explain up to 10% of the phenotypic variation. In addition, four, six, and three QTLs were identified associated with the PH, EH, and ER traits, respectively. Most of these QTLs decreased the phenotype value when they were from Zheng58, except for *qEH_Z6a* and *qEH_Z7a*, and six of them could explain up to 10% of the phenotypic variation.

### 3.3. Co-Localization of QTLs for Different Traits between the Two DH Populations

To determine the relationship of the six trait-related QTLs detected in different DH populations sharing a common parent Q1, we compared the physical interval corresponding to the two LOD interval for each QTL. Eleven chromosome regions contained overlapped QTLs for more than one trait, which were distributed on chromosome 1, 3, 5, 7, 8, 9, and 10 (Table 2, Figure 2). On chromosome 1, the region of 15.6–33.0 Mb includes two overlapped QTLs (*qPH_Y1a* and *qEH_Y1a*); the region of 35.5–172.2 Mb includes three overlapped QTLs (*qPH_Z1a*, *qEH_Z1a*, and *qER_Z1a)*; and the region of 221.1–277.0 Mb also includes three overlapped QTLs (*qAD_Y1a*, *qSD_Y1a*, and *qSD_Z1a*). On chromosome 3, the region of 6.7–11.9 Mb includes two QTLs (*qEH_Z3a* and *qER_Z3a*). On chromosome 5, the region of 20.3–165.9 Mb includes two QTLs (*qAD_Y5a* and *qSD_Y5a*); and the region of 147.4–217.3 Mb includes five QTLs (*qPH_Y5a*, *qEH_Y5a*, *qER_Y5a*, *qPH_Z5a*, and *qEH_Z5a*). On the chromosome 7, the region of 87.4–134.7 Mb includes two QTLs (*qEH_Y7a* and *qEH_Z7a*). On chromosome 8, the region of 110.5–163.8 Mb includes two QTLs (*qAD_Z8a* and *qSD_Z8a*). On chromosome 9, the region of 9.4–108.9 Mb includes two QTLs (*qPH_Y9a* and *qEH_Y9a*); and the region of 147.7–155.6 Mb includes two QTLs (*qPH_Z9a* and *qEH_Z9a*). On chromosome 10, the region of 14.4–129.5 Mb includes two QTLs (*qAD_Y10a* and *qPH_Z10a*). These overlapped chromosome regions could result from pleiotropy or linked genes. Among these genetic hotspots, the regions of 221.1–277.0 Mb on chromosome 1 (for flowering time traits), 147.4–217.3 Mb on chromosome 5 (for height traits), and 87.4–134.7 Mb on chromosome 7 (for height traits) were co-localized QTLs between the two DH populations, indicating that these regions could contribute to effects in different genetic backgrounds. We named these three co-localized genomic regions *qFT_YZ1a*, *qHT_YZ5a*, and *qHT_YZ7a*, respectively, and they will be the focus for further study.

## 4. Discussion

### 4.1. Relationship between Flowering Time and Height-Related Traits

Flowering time and height traits are two of the most studied traits in maize. Most studies indicate that these traits are closely correlated [36]. In this study, many correlations between flowering time and height traits were found to be significant, indicating that there could be pleiotropy or linked genes associated with these traits. In many crops, a series of genes have been validated to regulate both flowering time and plant height, for example, the rice genes *Ghd7*, *DTH8*, and *Hd1* [46,47,48], and the maize gene *Dwarf8* [49]. Additionally, some co-localized QTLs for these traits have also been identified, such as in maize [25], barley [50], and rice [51,52]. In our results, we found one significant co-localized genetic locus between flowering time and height traits, located at 14.4–129.5 Mb on chromosome 10 and contributing to AD and PH. These results provide genetic confirmation of the correlations between flowering time and height traits.

### 4.2. Potential Utilization of the Present QTLs

For any target trait, the QTL/gene that can be repeatedly identified across traits, populations, and environments is desirable for successful implementation of MAS breeding [31]. In the present study, we detected three consensus QTLs. Of these, the flowering time-related QTL *qFT_YZ1a* could explain 10.0–12.5% of AD and SD variation in both of the populations (Table 2), suggesting it may be a major QTL. The height trait-related QTL *qHT_YZ5a* contributed to 11.6–15.3% of PH and EH variation in both DH populations, which could also be a major QTL. Furthermore, we also found that these two consensus QTLs could contribute to effects in the three environments of the two populations (Table S2), indicating that they function stably. These consensus and stable and major QTLs may be considered priority candidates for MAS breeding. Another consensus QTL *qHT_YZ7a* could not be identified in all environments in the QZ population (Table S2), suggesting that environmental conditions and genetic background have a strong effect on its reliability. In addition to these consensus QTLs, other QTLs were detected only in individual populations, but some of them also contribute to a large effect. For example, *qAD_Y5a/qSD_Y5a* and *qAD_Y10a/qSD_Y10a* could explain up to 16.7% and 13.5% of AD and SD variation, respectively. *qPH_Y9a/qEH_Y9a*, *qPH_Z9a/qEH_Z9a*, *qPH_Z1a/qEH_Z1a/qER_Z1a*, and *qEH_Z3a/qER_Z3a* could contribute to more than 10% of PH and EH variation. At all of these loci, the early flowering or dwarf alleles were from the low-value parents Zheng58 or Ye478, indicating that they could improve the late flowering and higher height issues of Q1.

Decreasing height is usually a common target when applying exotic germplasm, especially tropical and sub-tropical germplasm, in breeding programs. However, plant height was positively correlated with grain yield [53], suggesting that we should decrease EH without affecting PH. Additionally, current high-density breeding targets need to similarly reduce ER traits [54]. This means ER may be more important than the PH and EH traits. In our results, the consensus QTL *qHT_YZ5a* was associated with the ER trait, further indicating its importance in improving height traits. Additionally, the population-specific QTLs *qEH_Z3a/qER_Z3a* and *qER_Z1a* could also explain up to 22.0% and 13.8% of ER variation, also suggesting their importance for improving height traits.

### 4.3. Comparison with QTLs Identified in Previous Studies

In the present study, we identified one flowering time-related consensus QTL *qFT_YZ1a*, located at 221.1–277.0 Mb on chromosome 1. Within the CI of *qFT_YZ1a*, the gene *id1* has been reported previously, and encodes a zinc finger protein and functions in late flowering [18]. This region also harbors the gene *Dwarf8*, which is implicated in both flowering time and plant height [49]. Interestingly, the orthologs gene of *Dwarf8* in wheat have contributed to the “green revolution” [24]. In addition, we also identified two other repeatedly identified QTLs for flowering time. Of these, *qAD_Z8a/qSD_Z8a* was located in the genomic region 110.5–163.8 Mb of chromosome 8, where *Vgt1* has been identified for late flowering [21], and *Zcn8* has been found to be a key photoperiod regulatory gene in maize [11]. The QTL *qAD_Y5a/ qSD_Y5a* was located in the genomic region 20.3–165.9 Mb of chromosome 5, where, to the best of our knowledge, no gene has been validated as being associated with flowering time.

For height traits, we identified two consensus QTLs between the two populations. Of these, *qHT_YZ5a* is located at bin 5.04–5.06 and is co-localized with four previously identified QTLs [30,31,32,33]. Additionally, within the CI of *qHT_YZ5a*, the gene *d9* has been validated as conferring dwarf characteristics and late flowering [38], and a major QTL *qPH5–1* also has been identified for the PH and EH of testcross performance and RILs per se [33]. In addition, another researcher also detected a dwarf allele in this region [55]. Interestingly, a lodging resistance-related QTL was found in this region, suggesting that lodging could be linked with height traits [56]. Another consensus QTL *qHT_YZ7a* located in the genomic region 87.4–134.7 Mb on chromosome 7 was significantly associated with only EH in the two DH populations. Within the CI of *qHT_YZ7a*, a major QTL for PH in normal and stress environments was detected [25]. In addition to these consensus QTLs, there were also four other repeatedly identified QTLs for height traits. Of these, *qPH_Y1a*/*qEH_Y1a* spanned the genomic region of 15.6–33.4 Mb on chromosome 1, where the gene *ZmRPH1* has been validated to be linked with PH and EH [39], and another similar QTL was also reported previously in this region [29]. The *qPH_Z1a/qEH_Z1a/qER_Z1a* overlapped with a QTL cluster conferring the PH, EH, and AD traits [57]. Another two repeatedly identified QTLs *qPH_Z3a/qEH_Z3a* and *qPH_Z9a/ qEH_Z9a* have not been found to have any major QTLs or genes associated with height traits to the best of our knowledge.

## 5. Conclusions

Flowering time and height are important agronomic traits in maize breeding. These traits are highly quantitative traits and are influenced by the environment. Thus, it is a challenge to improve them for phenotypic selection. In this study, we detected 34 QTLs conferring flowering time and height-related traits based on two DH populations, and 21 of these overlapped with at least one other QTL. Furthermore, ten QTLs had overlapping confidence intervals between the two DH populations and were integrated into three consensus QTLs. The flowering time-related consensus QTL *qFT_YZ1a* could explain 10–12.5% of the phenotypic variation for different traits in the two populations. This QTL is also located at a significant region of the previously detected flowering time QTL, which would be a major candidate region for future gene cloning and marker-assisted selection (MAS) breeding for flowering time. In addition, another consensus QTL *qHT_YZ5a* for height traits could explain 11.6–15.3% of phenotypic variation in different traits and populations. A number of QTLs were found in this region, suggesting that *qHT_YZ5a* could harbor novel major alleles. These two major consensus QTLs, in addition to other repeatedly detected QTLs, provide new insights for genetic improvement of flowering time and height-related traits.

## Figures and Tables

**Table 1:** Phenotypic summary of the two DH populations and their parents combined with the three environments.

Traits	Q1	Ye478	Zheng58	Population QY				Population QZ			
	Mean ± SD ^a^			Range ^b^	Mean ± SD	Skew. ^c^	Kurt. ^d^	Range	Mean ± SD	Skew.	Kurt.
AD (days)	89.1 ± 3.2	80.0 ± 2.1	81.9 ± 2.1	74.0–100.0	87.8 ± 4.6	−0.07	−0.17	72.0–99.0	84.3 ± 4.7	0.13	−0.47
SD (days)	94.0 ± 1.8	83.5 ± 1.8	85.2 ± 1.6	77.0–105.0	91.3 ± 4.7	−0.09	0.02	75.0–105.0	88.5 ± 5.3	0.05	−0.19
ASI (days)	4.9 ± 1.9	3.5 ± 0.4	3.4 ± 1.4	0–12.0	3.5 ± 1.9	0.78	0.66	−2.0–16.0	4.3 ± 2.3	0.84	1.60
PH (cm)	270.1 ± 8.9	174.5 ± 10.7	158.6 ± 9.7	123.0–310.0	206.7 ± 28.2	0.11	0.43	140.0–305.0	208.1 ± 28.4	0.16	−0.10
EH (cm)	120.3 ± 3.2	60.5 ± 3.8	50.6 ± 4.3	37.0–143.7	75.3 ± 18.1	0.32	0.12	34.3–157.0	71.0 ± 19.3	0.32	0.16
ER (%)	44.6 ± 2.6	34.2 ± 4.1	31.7 ± 3.2	19.8–57.9	36.3 ± 5.3	0.02	0.29	18.6–54.9	35.8 ± 6.3	−0.13	−0.17

^a^ standard deviation; ^b^ minimum value to maximum value; ^c^ skewness; ^d^ kurtosis. The traits of AD, SD, ASI, PH, EH, and ER represent days to anthesis, days to silking, anthesis–silking interval, plant height, ear height, and EH/PH ratio, respectively. The population QY represents the DH population constructed by Q1 and Ye478, and the population QY represents the DH population constructed by Q1 and Zheng58.

**Table 2:** QTLs for the six flowering time and height-related traits in the two DH populations.

Pop.	QTL Name	Chr.	Peak (cM)	LOD	Add.	R2 (%)	2-LOD Interval (cM)	Range (Mb)	Trait
QY	*qPH_Y1a*	1	40.2	4.2	−6.7	8.8	36.6–50.4	15.6–33.4	PH
	*qEH_Y1a*	1	44.3	3.2	−3.4	7.0	41.3–49.6	22.6–33.0	EH
	*qAD_Y1a*	1	173.9	6.5	−1.0	12.5	165.7–185.4	234.3–277.0	AD
	*qSD_Y1a*	1	173.9	5.3	−1.0	10.7	162.4–184.3	232.2–272.1	SD
	*qSD_Y3a*	3	35.3	3.5	−0.8	6.8	28.7–36.1	4.9–6.5	SD
	*qSD_Y5a*	5	62.3	8.0	−2.0	16.7	54.1–71.3	20.3–165.9	SD
	*qAD_Y5a*	5	66.5	4.3	−0.8	8.3	54.1–71.1	20.3–165.9	AD
	*qPH_Y5a*	5	91.9	7.2	−9.0	15.3	74.6–96.0	165.9–203.8	PH
	*qEH_Y5a*	5	102.5	5.6	−4.8	13.3	91.4–112.9	195.8–214.0	EH
	*qER_Y5a*	5	117.3	3.0	−1.0	7.8	106.6–121.4	207.7–217.3	ER
	*qASI_Y5a*	5	130.4	4.1	1.2	11.7	128.8–140.3	218.9–221.1	ASI
	*qPH_Y7a*	7	44.3	3.1	−5.8	6.5	42.6–50.0	24.7–45.7	PH
	*qEH_Y7a*	7	70.7	3.3	−3.5	7.7	63.2–80.6	119.5–134.7	EH
	*qEH_Y9a*	9	37.7	5.1	−4.5	12.0	26.5–48.4	9.4–21.6	EH
	*qPH_Y9a*	9	52.0	6.0	−8.4	14.0	43.7–65.6	16.8–108.9	PH
	*qAD_Y10a*	10	34.5	4.3	−0.8	8.2	31.7–44.9	14.4–129.5	AD
	*qSD_Y10a*	10	56.0	6.3	−1.1	13.5	45.1–59.9	129.5–142.3	SD
QZ	*qER_Z1a*	1	90.0	7.9	−1.7	13.8	73.8–96.0	35.5–71.2	ER
	*qEH_Z1a*	1	94.2	6.6	−4.6	11.2	83.4–107.2	51.4–85.1	EH
	*qPH_Z1a*	1	109.0	5.0	−5.9	7.7	99.7–125.5	72.9–172.2	PH
	*qSD_Z1a*	1	189.6	5.0	−0.9	10.0	176–197.0	221.1–240.4	SD
	*qER_Z1a*	1	239.6	3.0	−1.0	4.8	235.3–250.5	279.6–285.5	ER
	*qEH_Z3a*	3	60.6	4.1	−3.5	6.6	49.6–65.5	6.7–11.9	EH
	*qER_Z3a*	3	60.6	12.0	−2.1	22.0	55.6–63.7	8.5–10.5	ER
	*qAD_Z4a*	4	99.8	6.4	−0.8	11.3	83.4–104.9	158.2–181.5	AD
	*qPH_Z5a*	5	111.7	7.5	−7.4	12.4	99.3–123.7	147.4–175.0	PH
	*qEH_Z5a*	5	128.0	6.8	−4.7	11.6	116.7–138.5	168.6–199.3	EH
	*qEH_Z6a*	6	153.7	3.0	2.9	4.4	146.5–160.7	166.8–169.7	EH
	*qEH_Z7a*	7	68.0	3.2	3.1	5.1	56.8–78.7	87.4–126.7	EH
	*qSD_Z8a*	8	85.5	3.8	−0.7	7.3	79.4–96.5	110.5–156.3	SD
	*qAD_Z8a*	8	97.8	3.2	−0.6	5.6	80.9–99.3	112.4–163.8	AD
	*qPH_Z9a*	9	97.9	8.9	−9.2	14.7	90.0–108.8	147.7–155.6	PH
	*qEH_Z9a*	9	97.9	4.2	−4.0	6.8	89.7–105.9	147.7–155.2	EH
	*qPH_Z10a*	10	29.0	3.1	−4.8	4.8	23.5–30.3	17.4–69.0	PH

“Pop.” represents the population from which the QTL was detected; “Chr.” is the chromosome; “LOD” is logarithm of odds; “Add.” represents additive effect of QTL; “R2” is phenotypic variation explained by each QTL; “2 LOD interval” represents confidence interval that defines the 2 LOD interval of the QTL; “Range” represents the physical interval of the QTL distributed in the chromosome according to B73 RefGen_v4.

## Data Availability

Not applicable.

## Supplementary Materials

The following materials are available online at https://www.mdpi.com/article/10.3390/plants10081585/s1, Table S1: Analysis of variance for the six flowering time and height traits of two DH populations over three environments. Table S2: QTLs for the six flowering time and height related traits in the two DH populations and three environments. Figure S1: The performance of three parents around tasseling period.
