# Supplementary material for: Dissecting the Genetic Basis of Flowering Time and Height Related-Traits Using Two Doubled Haploid Populations in Maize
Source: Plants (Basel). 2021 Jul 31;10(8):1585. doi: 10.3390/plants10081585 (PMC8399143; doi:10.3390/plants10081585)
Supplement: Supplementary file 1 [file plants-10-01585-s001.zip › Table S1.pdf]

Table S1 Analysis of variance for the six flowering time and height traits of two DH populations over three environments

Source of variation	PH	EH	ER	AD	SD	ASI
Population QY:						
Genotype	<.0001	<.0001	<.0001	<.0001	<.0001	<.0001
Environment	<.0001	<.0001	<.0001	<.0001	<.0001	<.0001
Replicate	0.0860	0.4300	0.7470	0.2260	0.2370	0.7980
Genotype*Environment	<.0001	<.0001	<.0001	<.0001	<.0001	<.0001
H^2 (%)	87.2	82.0	70.7	75.5	75.3	28.5
Population QZ:						
Genotype	<.0001	<.0001	<.0001	<.0001	<.0001	<.0001
Environment	<.0001	<.0001	<.0001	<.0001	<.0001	<.0001
Replicate	0.0506	0.0292	0.1330	0.5300	0.6610	0.3420
Genotype*Environment	<.0001	<.0001	<.0001	<.0001	<.0001	<.0001
H^2 (%)	83.6	82.6	82.8	81.8	71.4	39.9
