# Supplementary material for: Dissecting the Genetic Basis of Flowering Time and Height Related-Traits Using Two Doubled Haploid Populations in Maize
Source: Plants (Basel). 2021 Jul 31;10(8):1585. doi: 10.3390/plants10081585 (PMC8399143; doi:10.3390/plants10081585)
Supplement: Supplementary file 1 [file plants-10-01585-s001.zip › Table S2.pdf]

Table S2 QTLs for the six flowering time and height related traits in the two DH populations and three environments

Pop. [a]	Chr. [b]	Pos. (cM) [c]	LOD [d]	Add. [e]	R2 (%) [f]	2 LOD (cM)[g]	Range (Mb)[h]	Trait [i]	Env. [j]
QY	1	2.8	3.1	-6.8	6.9	0–9.0	0–6.2	PH	17H
QY	1	40.2	3.3	-7.8	7.2	33.7–42.6	15.7–23.3	PH	16H
QY	1	40.2	6.6	-6.2	15.8	34.2-45.8	15.7-27.3	EH	16H
QY	1	43.5	5.0	-1.7	14.0	35.0–48.0	15.7–29.6	ER	16H
QY	1	156.7	5.7	-1.3	14.6	139.6–161.3	205.2–231.4	AD	17Z
QY	1	156.7	5.7	-1.3	14.6	139.6-161.3	205.2-231.4	SD	17Z
QY	1	159.9	3.6	-1.7	9.9	152.0–161.6	223.3-232.2	ER	17H
QY	1	173.1	4.0	-1.1	7.9	170.6–175.5	241.0–249.5	AD	16H
QY	1	173.1	4.2	-1.2	8.2	170.6-175.5	241.0-249.5	SD	16H
QY	1	173.9	5.5	-1.4	10.3	172.2–178.4	242.8–259.3	AD	17H
QY	1	173.9	7.3	-1.6	14.2	172.2-184.2	241.0-272.1	SD	17H
QY	2	97.8	3.3	1.0	8.2	85.8-100.3	198.3-218.4	SD	17Z
QY	3	165.9	3.1	2.0	9.3	156.4–175.0	227.3-231.7	ER	17Z
QY	4	11.2	3.2	-1.8	9.9	3.3–22.2	1.5-7.2	ER	17Z
QY	4	13.2	4.6	-6.9	13.2	5.7-21.4	1.8-7.22	EH	17Z
QY	5	64.3	5.2	-2.6	11.4	61.5-65.3	33.0-55.9	SD	16H
QY	5	65.3	5.0	-2.3	10.1	57.4–65.6	24.2–55.9	AD	16H
QY	5	66.5	7.6	-1.5	14.9	54.6–70.1	20.4–84.4	AD	17H
QY	5	66.5	6.0	-1.4	11.4	65.6-70.6	42.9-165.9	SD	17H
QY	5	87.8	6.6	-11.6	15.0	74.5–95.8	170.0–203.0	PH	16H
QY	5	88.6	9.0	-7.7	22.5	86.1-96	183.3-203.8	EH	16H
QY	5	88.6	3.8	-1.5	10.2	83.8–99.3	181.7-205.6	ER	16H
QY	5	91.9	5.0	-10.1	13.0	81.2–97.6	179.5–204.6	PH	17Z
QY	5	91.9	3.5	-5.5	8.7	91.4-94.3	195.9-201.6	EH	17H
QY	5	102.5	6.4	-10.1	14.6	91.6–106.8	197.5–209.7	PH	17H
QY	5	102.5	5.6	-6.6	13.5	101.7-109.9	207.4-210.7	EH	17H
QY	7	32.0	4.8	-1.3	9.6	28.7–38.5	7.5–14.7	AD	16H
QY	7	32.0	4.8	-1.3	9.6	28.7-38.5	7.4-14.7	SD	16H
QY	7	41.0	3.2	-7.6	6.9	36.1–42.6	11.2–30.7	PH	16H
QY	7	41.0	4.1	-7.9	8.9	36.1–42.6	11.2–30.7	PH	17H
QY	7	70.7	3.3	-4.9	7.9	58.3-79	111.2-131.9	EH	17H
QY	7	70.7	5.6	-14.8	17.9	69.5-74.5	121.6-127.4	EH	17Z
QY	9	37.7	3.3	-4.3	7.3	26.7-45.9	9.4-18.5	EH	16H
QY	9	37.7	5.4	-6.4	13.0	33.6-48.0	12.3-20.2	EH	17H
QY	9	52.0	5.5	-10.4	12.9	49.9–73.2	21.6–136.4	PH	16H
QY	9	55.0	6.5	-10.8	14.8	50.4–65.3	22.5–108.9	PH	17H
QY	9	76.7	6.3	-12.7	17.5	63.0–86.3	98.5–154.7	PH	17Z
QY	10	34.5	4.7	-1.2	8.7	33.4–44.6	19.9–13.0	AD	17H
QY	10	34.5	5.6	-1.4	16.1	34.1–44.2	22.5–12.2	AD	17Z

Continued table

Pop. [a]	Chr. [b]	Pos. (cM) [c]	LOD [d]	Add. [e]	R2 (%) [f]	2 LOD (cM)[g]	Range (Mb)[h]	Trait [i]	Env. [j]
QY	10	34.5	3.7	-1.1	6.6	31.2-44.7	14.5-122.4	SD	17H
QY	10	34.5	5.5	-1.3	15.5	33.6-44.1	19.9-122.4	SD	17Z
QZ	1	84.3	6.5	-2.1	12.5	83.7–95.2	52.7-71.2	ER	17H
QZ	1	84.9	9.8	-2.4	18.9	74.8–92.1	38.2-66.8	ER	16H
QZ	1	90.5	6.1	-6.0	12.0	83.7-97.9	52.8-76.1	EH	16H
QZ	1	94.2	4.1	-8.3	7.1	91.1–96.0	59.1–72.0	PH	17H
QZ	1	94.2	8.5	-7.8	15.2	83.7-96	52.8-71.2	EH	17H
QZ	1	100.3	7.8	-7.4	14.1	97.9-106.3	72.9-80.9	EH	17H
QZ	1	103.3	5.4	-5.7	11.5	100.3-106.3	76.1-80.9	EH	16H
QZ	1	115.2	3.1	-5.8	5.0	100.0–123.2	77.4–133.6	PH	17Z
QZ	1	145.5	3.8	-0.5	9.1	141.8–148	183.9-200.4	ASI	17Z
QZ	1	156.0	3.0	-5.9	5.3	147.4–162.4	197.0–210.2	PH	16H
QZ	1	187.6	3.2	-0.9	5.7	173.4–198.5	221.0–240.0	AD	17H
QZ	1	187.6	3.2	-0.9	5.7	173.4-198.5	221.1-240.4	SD	17H
QZ	1	223.5	3.8	-0.9	6.5	215.1–226.6	257.9–274.4	AD	16H
QZ	1	223.5	3.8	-0.9	6.5	215.1-226.6	257.9-274.8	SD	16H
QZ	1	225.4	6.3	-1.1	13.9	220.2–229.2	263.3–275.8	AD	17Z
QZ	1	225.4	6.3	-1.1	13.9	220.2-229.2	261.8-275.8	SD	17Z
QZ	1	227.2	3.7	-5.0	7.2	226.6-239.3	274.4-281.8	EH	17Z
QZ	2	30.3	4.4	2.2	8.3	21.4–35.7	4.39-9.7	ER	17H
QZ	2	56.2	4.3	1.5	7.6	44.9–80.2	12.0-22.9	ER	16H
QZ	3	57.9	5.9	-6.4	10.4	45.5-65.5	5.9-11.9	EH	17H
QZ	3	59.9	4.3	-5.1	7.8	55-70.2	8.5-12.1	EH	17Z
QZ	3	60.6	6.9	-1.9	12.8	56.3–65.4	8.5-11.5	ER	16H
QZ	3	60.6	8.1	-2.4	16.1	54.5–71.2	8.05-12.5	ER	17H
QZ	3	60.6	8.8	-2.6	18.8	56.0–63.7	8.5-10.5	ER	17Z
QZ	3	64.3	5.3	-5.7	10.3	53.6-71.2	6.9-12.5	EH	16H
QZ	3	128.8	3.6	1.8	7.2	118.4–141.1	134.1-156.8	ER	17Z
QZ	3	208.0	3.8	-0.7	8.6	205.9–214.1	218.2-220.2	ASI	16H
QZ	4	31.2	3.8	-6.7	6.7	25.7–38.4	5.5–10.1	PH	16H
QZ	4	39.9	4.0	-0.8	6.9	34.9–46.7	6.0–12.3	AD	16H
QZ	4	39.9	4.0	-0.8	6.9	34.9-46.7	6.0-12.6	SD	16H
QZ	4	100.8	5.3	-1.2	10.9	96.1–102.8	175.8–180.3	AD	17H
QZ	4	100.8	5.3	-1.2	10.9	96.1-102.8	175.9-180.3	SD	17H
QZ	5	110.7	7.2	-9.1	12.4	100.2–118.1	154.2–171.2	PH	17Z
QZ	5	114.2	4.4	-8.6	8.2	100.2–123.3	154.2–174.7	PH	17H
QZ	5	116.9	3.1	-4.4	5.6	116.7-118.1	168.6-171.2	EH	17Z
QZ	5	117.9	7.5	-7.3	13.2	107.8-131.2	162.4-191.2	EH	17H
QZ	5	133.0	4.1	-5.1	7.3	130.5-138.4	190.1-199.3	EH	17Z
QZ	5	133.0	3.7	-1.6	7.4	118.7–139.7	171.2-200.6	ER	17Z

Continued table

Pop. [a]	Chr. [b]	Pos. (cM) [c]	LOD [d]	Add. [e]	R2 (%) [f]	2 LOD (cM)[g]	Range (Mb)[h]	Trait [i]	Env. [j]
QZ	5	141.0	3.5	-4.8	6.7	130.5-145.9	190.1-204.6	EH	16H
QZ	6	15.5	4.5	-0.9	10.4	9.3–31.0	25.6-95.9	ASI	16H
QZ	6	63.0	3.3	0.6	8.8	46.4–66.1	107.2-121.8	ASI	17H
QZ	6	113.8	5.3	1.1	12.2	112.3–116.5	156.8-159.7	ASI	17H
QZ	6	152.7	3.5	4.7	5.7	145.9-155.8	166.9-168.7	EH	17H
QZ	8	85.5	5.1	-1.2	9.5	80.7–99.2	112.4–149.5	AD	17H
QZ	8	85.5	5.1	-1.2	9.5	80.7-86.7	112.4-143.0	SD	17H
QZ	8	97.8	4.7	-0.9	8.2	89.5–99.7	147.7–150.4	AD	16H
QZ	8	97.8	4.7	-0.9	8.2	89.5-99.7	146.0-164.5	SD	16H
QZ	8	97.8	4.3	-1.1	8.0	96.6-99.2	156.3-163.8	SD	17H
QZ	8	101.5	5.1	-1.2	11.1	90.6–105.2	149.5–166.5	AD	17Z
QZ	8	101.5	5.1	-1.2	11.1	96.9-105.2	155.4-166.9	SD	17Z
QZ	9	97.1	4.9	-9.3	9.3	88.3–99.6	147.7–154.3	PH	16H
QZ	9	97.9	6.4	-11.7	11.6	90.9–99.8	152.9–154.3	PH	17H
QZ	9	97.9	5.5	-9.1	9.2	86.6–99.1	147.7–154.0	PH	17Z
QZ	10	29.0	3.4	-4.9	5.7	22.3-33.6	15.2-79.4	EH	17H

[a] population; [b] chromosome; [c] the peak position of the QTL; [d] logarithm of odds; [e] addition effect; [f] phenotypic variation explained by each QTL; [g] confidence interval that defines 2-LOD interval of QTL; [h] physical interval of QTL distributed in chromosome according to B73 RefGen_v4; [i] QTL was detected for which trait; [j] QTL was detected which environments.
