# Supplementary figures and images for: Dissecting the Genetic Basis of Flowering Time and Height Related-Traits Using Two Doubled Haploid Populations in Maize
Source: Plants (Basel). 2021 Jul 31;10(8):1585. doi: 10.3390/plants10081585 (PMC8399143; doi:10.3390/plants10081585)

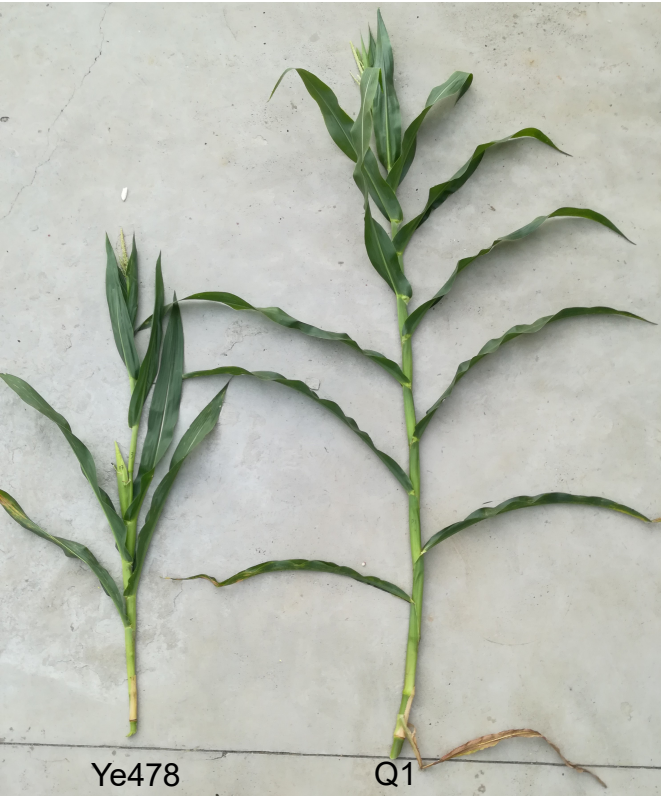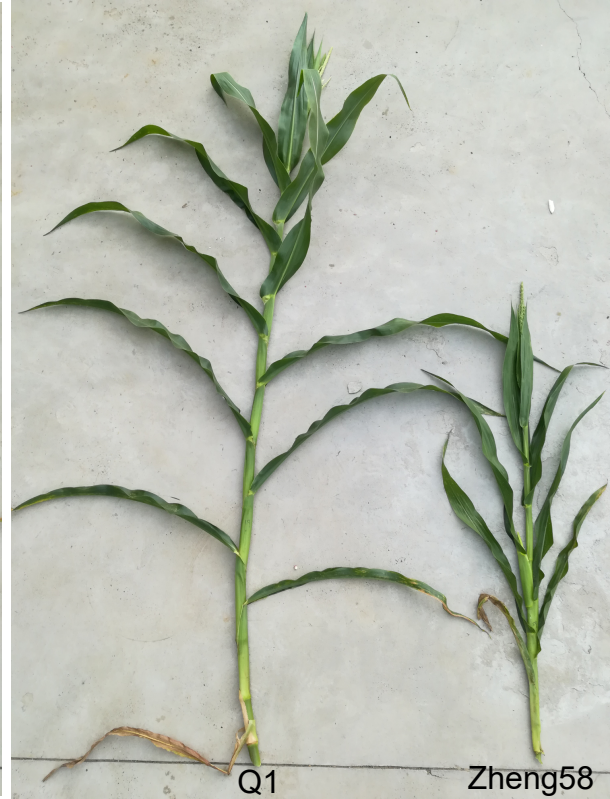

Supplement: Supplementary file 1 [file plants-10-01585-s001.zip › Figure S1.pdf]
